# Growth and feeding of deep-sea coral *Lophelia pertusa* from the California margin under simulated ocean acidification conditions

**DOI:** 10.7717/peerj.5671

**Published:** 2018-09-27

**Authors:** Carlos E. Gómez, Leslie Wickes, Dan Deegan, Peter J. Etnoyer, Erik E. Cordes

**Affiliations:** 1 Department of Biology, Temple University, Philadelphia, PA, United States of America; 2 JHT, Inc, Orlando, FL, United States of America; 3 Thrive Blue, LLC, Denver, CO, United States of America; 4 NOAA National Center for Coastal Ocean Science, Charleston, SC, United States of America

**Keywords:** Deep-sea, Carbonate saturation, Climate change, Ocean acidification

## Abstract

The global decrease in seawater pH known as ocean acidification has important ecological consequences and is an imminent threat for numerous marine organisms. Even though the deep sea is generally considered to be a stable environment, it can be dynamic and vulnerable to anthropogenic disturbances including increasing temperature, deoxygenation, ocean acidification and pollution. *Lophelia pertusa* is among the better-studied cold-water corals but was only recently documented along the US West Coast, growing in acidified conditions. In the present study, coral fragments were collected at ∼300 m depth along the southern California margin and kept in recirculating tanks simulating conditions normally found in the natural environment for this species. At the collection site, waters exhibited persistently low pH and aragonite saturation states (Ω_arag_) with average values for pH of 7.66 ± 0.01 and Ω_arag_ of 0.81 ± 0.07. In the laboratory, fragments were grown for three weeks in “favorable” pH/Ω_arag_ of 7.9/1.47 (aragonite saturated) and “unfavorable” pH/Ω_arag_ of 7.6/0.84 (aragonite undersaturated) conditions. There was a highly significant treatment effect (*P* < 0.001) with an average% net calcification for favorable conditions of 0.023 ± 0.009% d^−1^ and net dissolution of −0.010 ± 0.014% d^-1^ for unfavorable conditions. We did not find any treatment effect on feeding rates, which suggests that corals did not depress feeding in low pH/ Ω_arag_ in an attempt to conserve energy. However, these results suggest that the suboptimal conditions for *L. pertusa* from the California margin could potentially threaten the persistence of this cold-water coral with negative consequences for the future stability of this already fragile ecosystem.

## Introduction

Global mean values of atmospheric carbon dioxide (CO_2_) have increased dramatically since pre-industrial times from 280 ppm (parts per million) to about 405 ppm in 2017. This increase in atmospheric CO_2_ is unprecedented in at least the past 650 thousand years during the last four glacial cycles (Siegenthaler et al., 2005). As the oceans come into equilibrium with the atmosphere, there is an alteration of carbonate chemistry with an increase in the concentration of hydrogen ions and a decrease in carbonate saturation states (Ω) (Kleypas et al., 1999; Caldeira & Wickett, 2003). The aragonite saturation horizon (ASH) is a boundary in the water column at which the carbonate saturation state is equal to 1 (Ω = 1), and is found at two different depths corresponding to the different carbonate polymorphs of aragonite (Ω_arag_) and calcite (Ω_cal_). If the water is supersaturated (Ω >  1) calcification is favored, whereas if it is undersaturated (Ω <  1) dissolution is favored over calcification (Gattuso et al., 1998; Langdon & Atkinson, 2005). In a biological context, the lower the saturation state, the more energy required for calcification. Although global ocean calcium carbonate saturation states remain above 1 for most shallow portions of the ocean, models forecast a significant shoaling of the saturation horizon (ASH) by mid-century (Orr et al., 2005). This will have negative impacts to deep-sea ecosystems, since deep-water organisms live in habitats that already experience lower saturation states than their shallower counterparts.

*Lophelia pertusa* is the most well-known cold-water coral (CWC) with cosmopolitan distribution at depths normally between 40 and 800 m (Roberts et al., 2009). Hard substrate, local topography, temperature, current flow, and food supply have been cited as factors that can affect its distribution (Davies & Guinotte, 2011; Georgian, Shedd & Cordes, 2014). Aragonite saturation (Ω_arag_) has also been proposed as an important factor due to the evidence that >95% of CWCs are distributed in places where the saturation of calcium carbonate is above 1 (Ω > 1) (Guinotte et al., 2006). Nevertheless, recent deep-sea explorations have led to new observations of scleractinian corals at aragonite undersaturation, such as the Central North Pacific (Baco et al., 2017, ASH < 550 m), Chilean Fjiords (Fillinger & Richter, 2013; Jantzen et al., 2013, ASH < 200 m), and the Central South Pacific (Thresher et al., 2011, ASH < 1,000 m). The mechanisms underlying this apparent ability to live under conditions that are not favorable for calcification remain unknown, since experimental studies with coral species from those places are not common. However, the capacity to alter the internal carbonate chemistry in favor of calcification has been proposed as a plausible explanation (Raybaud et al., 2017). Due to the significant ecological role of these deep-water habitats, understanding the complex relationship among the biology, physiology, and ecology of cold-water corals with their realized distribution is of prime importance.

The potential effects of future levels of ocean acidification on the physiological performance of CWCs have shown some contrasting evidence, nevertheless, there is good agreement about its potential negative effects (Maier et al., 2009; Hennige et al., 2015; Georgian et al., 2016a; Kurman et al., 2017). On one hand, Form & Riebesell (2012) studied the short and long-term response of *Lophelia pertusa* from the North Atlantic grown under different levels of pH and aragonite saturation states (Ω_arag_). They found that in the long-term, corals were able to maintain and even increase calcification rates under high CO_2_ conditions. Büscher, Form & Riebesell (2017) found no significant effects of low Ω_arag_ on growth rates of *L. pertusa* in the long-term, although they observed decreased calcification. On the other hand, Kurman et al. (2017) showed significant detrimental effects of ocean acidification on calcification of *L. pertusa* in the long-term (6 months), and that in the short term (∼2 weeks) some of the fragments experienced net dissolution rates at undersaturated levels of aragonite, although these differences were not significant due to a high variability in the response. Georgian et al. (2016a) found that different populations of *L. pertusa* from the Gulf of Mexico and Norwegian Skagerrak had different responses at undersaturated levels of aragonite, with the Norwegian population capable of elevating feeding rate to maintain growth at low pH, while populations of the Gulf of Mexico reduced feeding rate and calcification rates, presumably to save energy. Moreover, Hennige et al. (2015) showed that *L. pertusa* can survive undersaturation but with the cost of losing framework stability.

Upwelling systems are characterized by naturally higher CO_2_ concentrations, and lower pH and Ω_arag_ than non-upwelling areas (Feely et al., 2004). These are also areas with high primary productivity, which has been shown to affect the structure and composition of the benthic fauna, including increasing deep-sea coral diversity (Davies et al., 2008; Jansen et al., 2018). The California Current System (CCS) is a key Pacific Ocean current that moves equatorward throughout the year along the west coast of North America (Lynn & Simpson, 1987), and is one of the four major Eastern Boundary Upwelling Systems, which has been associated with ocean acidification processes (Feely et al., 2008; Gruber et al., 2012). Modeled data have suggested that since preindustrial times, the CCS has already experienced a dramatic decrease in pH of ∼0.1 and Ω_arag_ of ∼0.4, with the current surface mean pH and Ω_arag_ of 7.95 ±  0.04 and 1.67 ± 0.16 respectively (Gruber et al., 2012). This is especially relevant given the ecological, biological, and economical importance of the CCS (Chan et al., 2008; Barton et al., 2015). In summer, when the system experiences strong upwelling, models predict undersaturation throughout the water column for coastal as well as off-shore waters (Gruber et al., 2012).

In order to understand the physiological response of *L. pertusa* from the California margin, we conducted a series of short-term experiments to examine the calcification and feeding behavior of corals collected from the Southern California Bight. Fragments of *L. pertusa* were grown under controlled conditions that simulated the present-day scenario *in situ* (aragonite undersaturation) hereafter referred to as “unfavorable” vs. saturated aragonite conditions (corresponding to >95% of the present-day global distribution of *L. pertusa*) hereafter referred to as “favorable”. We hypothesized that corals growing in unfavorable conditions (Ω_arag_ < 1) will calcify less than corals grown in favorable conditions (Ω_arag_ > 1), but we still expect calcification rates to be positive (accretion > erosion). The present conditions in the California Current System are similar to those that are expected to occur by the end of the century in other areas (Gruber et al., 2012), so the information regarding how these corals are responding is crucial to understanding the whole ecosystem response in the CCS and beyond.

## Material and Methods

### Sample collection

**Figure 1:**
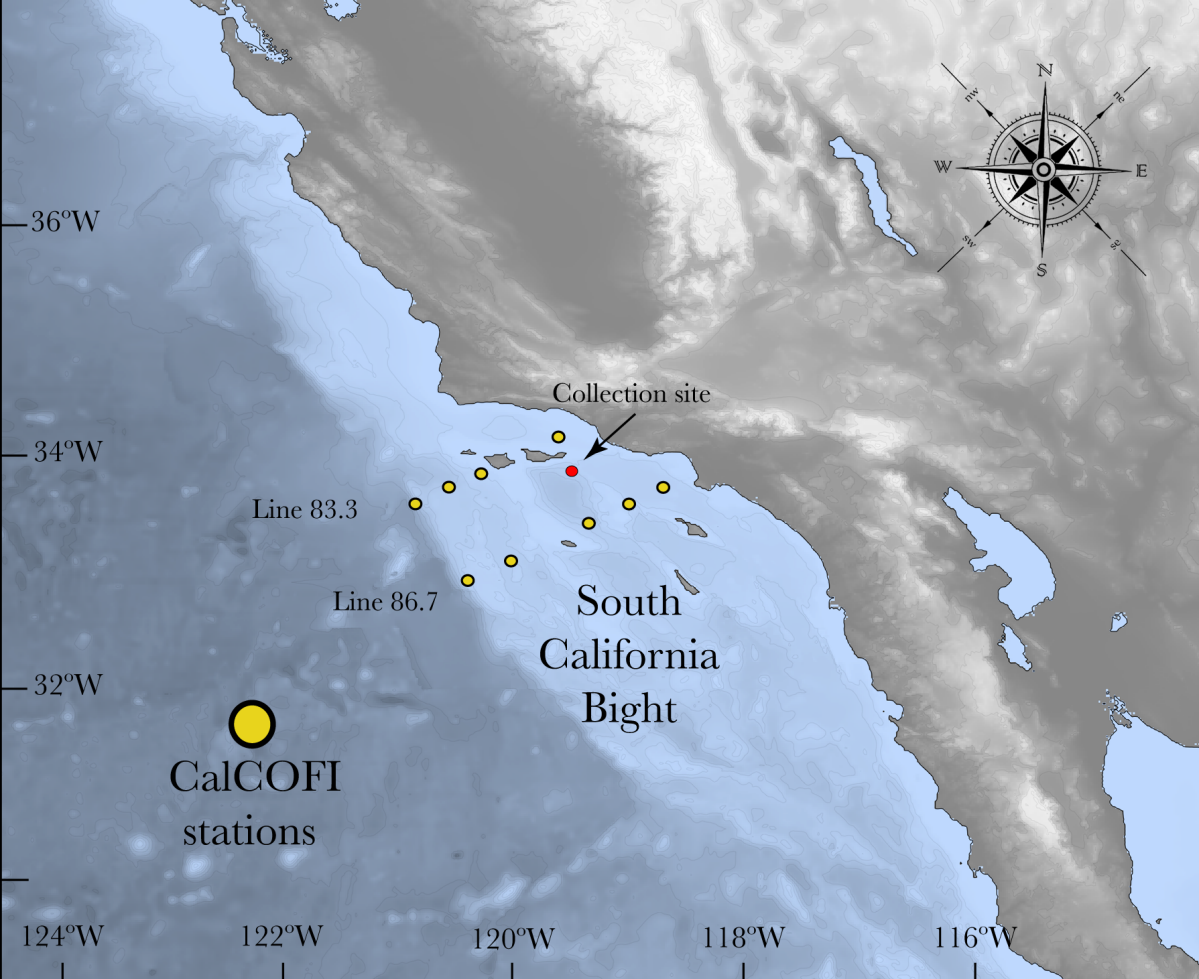
Map of the South California Bight showing the sampling station (red dot). Yellow dots refer to the CalCOFI stations used to complement the characterization of the carbonate chemistry of the area where *L. pertusa* was collected.

*Lophelia pertusa* fragments were collected in the Southern California Bight (SCB) between April and May 2015 (33°55′7.6794″N; 119°28′18.84″W) at ∼300 m depth under permit number CINMS-2015-002, Feb 4 2015 by NOAA office of National Marine Sanctuaries in support to the project “Climate Vulnerability Assessment for Deep-Sea Corals Ecosystems in California” in order to conduct research activities in the Channel Islands National Marine Sanctuary to take resources using an ROV, and specifically to collect biological specimens (Caldow, Etnoyer & Kracker, 2015; Fig. 1). After collection, all live corals were kept in natural seawater at an ambient temperature of ∼9 °C using insulated containers. They were transported to the lab in Charleston, SC where they were maintained for over 1 year in a 550 L recirculating system containing custom-made artificial seawater that simulates the natural seawater conditions. Corals were maintained at a temperature of ∼9 °C , salinity 35, and total alkalinity (A_T_) ∼2,300 µmol kg^−1^. The corals were fed every other day with a mixture of zooplankton-phytoplankton (Fauna Marin^®^, Holzgerlingen, Germany) and artificial Marine Snow^®^ (Two Little Fishies, Miami Gardens, FL, USA).

### Seawater chemistry at the collection site

The California Current System (CCS) is an offshore water mass characterized by waters with low temperature and salinity that flow equatorward along the west coast of the US (Lynn & Simpson, 1987). North Pacific Ocean water masses that influence this system are relatively low in pH and hence carbonate saturation (Harris, DeGrandpre & Hales, 2013; Hauri et al., 2013). Due to the periodic upwelling of deeper water masses, the CCS experiences frequent periods of aragonite undersaturation, which are similar to the future effects of ocean acidification in other areas (Feely et al., 2008; Hauri et al., 2009; Gruber et al., 2012; Barton et al., 2015). In order to assess the carbonate chemistry near the *L. pertusa* coral collection location, two inorganic carbon parameters (pH_T_ and A_T_) were measured from two different sources: (1) Discrete water samples obtained from seven CTD casts (August 2014, March 2015, August 2015) and (2) CTD data from the California Cooperative Oceanic and Fisheries Investigation program (CalCOFI) stations that were sampled forming a grid within the northern part of the Southern California Bight (SCB) that includes the sample collection sites (Fig. 1). Refer to CalCOFI.org for more information about the grid and stations.

The CTD rosette was deployed in the area of major coral aggregations at the Piggy Bank and Footprint coral sites (33°55′7.32″N; 119°28′19.5594″W and 33°57′48.95″N; 119°29′22.2″W respectively) in order to characterize the carbonate system in the environment in which *L. pertusa* grows (Caldow, Etnoyer & Kracker, 2015). The CTD rosette was composed of Niskin bottles and a Sea-Bird CTD unit that varied according to the year of collection. In 2014, the CTD rosette was composed of four Niskin bottles with CTD Sea-Bird SBE 19+, in March 2015 it was composed of 12 Niskin bottles and a Sea-Bird SBE 9, and for August 2015 it was composed of six Niskin bottles and Sea-Bird SBE 19+. Soon after collection, water samples were transferred from the Niskin to an empty and clean Nalgene HDPE bottle (250 ml) using silicone tubing, making sure no bubbles were added to the sample and filled up to the top without headspace (Dickson, Sabine & Christian, 2007). Water samples were brought to room temperature and the pH_T_ (total scale) measurement was performed in replicate within 4 h of collection (Dickson, Sabine & Christian, 2007). Immediately after pH_T_ measurement, 50 µL of a saturated solution of mercuric chloride was added to poison the sample and prevent alterations of the carbonate chemistry by biological activity (Dickson, Sabine & Christian, 2007). Samples were stored in a cool and dark location until further analysis for total alkalinity (A_T_) at NOAA’s Center for Coastal Environmental and Biomolecular Research. The other carbonate parameters were calculated from pH_T_ and A_T_ using the software CO2calc (Robbins et al., 2010).

Additionally, CTD data (salinity, temperature, depth and oxygen) from CalCOFI were used to provide a more complete spatial and temporal characterization of the seawater chemistry variability of the South California Bight (SCB) for 2015, the year of collection. CalCOFI is one of the most complete, large-scale, and high-quality hydrographic sampling grids, and has been conducted since 1949 along the California Current from San Francisco to Baja California. The grid consists of a series of stations in parallel lines extending perpendicular to the coast (Bograd, Checkley & Wooster, 2003). Since 1964, CTD casts have been taken 3–4 times a year from the surface to a depth of 500 m (Lynn & Simpson, 1987). In the Southern California Bight (SCB), stations are approximately 30 km separated from each other, with a relatively high occupancy compared to other stations in the grid (Lynn & Simpson, 1987). Using the approximations given by Alin et al. (2012) specifically developed for the SCB from the CalCOFI data, we approximated the A${}_{\mathrm{T}}^{\mathrm{es}}$ and pH${}_{\mathrm{T}}^{\mathrm{es}}$ in a grid of approximately 100 km^2^ that encompasses the area where *L. pertusa* were collected in SCB (Fig. 1). From the approximated A${}_{\mathrm{T}}^{\mathrm{es}}$ and pH^es^, we obtained the other carbonate parameters such as [pCO_2_], [HCO_3_^−^], [CO_3_^−2^] and [Ω_arag_] using the software CO2Calc (Robbins et al., 2010) with the dissociation constants for boric acid and K_1_ and K_2_ from Leuker, Dickson & Keeling (2000), KHSO_4_ from Dickson (1990), total boron from Lee et al. (2000) and pH on the total scale (pH_T_).

CTD data from 18 points that represented 10 different stations in the grid were selected, from which A${}_{\mathrm{T}}^{\mathrm{es}}$ and pH^es^ were approximated (CalCOFI Line 86.7: stations 35–40–45–55–60 and Line 83.3: stations 40–42–51–55–60). Each station was comprised of 4 points spanning one year of sampling (November 2014–March 2015–August 2015–November 2015) that were averaged in order to get one value per station with a measure of variability. Since it is known that the shallower portions (0–80 m) are more variable (Juranek et al., 2009; Alin et al., 2012), and *L. pertusa* has not been found there, we excluded these depths and the analysis was performed from 80–400 m.

**Table 1:** Summary of seawater carbonate chemistry conditions at Piggy Bank and experimental tanks. Seawater carbonate chemistry conditions at Piggy Bank where the samples were collected and carbonate chemistry conditions in the experimental tanks. T1 trough T3 refers to the treatment conditions. Values are given as mean ± SD. Total alkalinity (A_T_), pH on total scale (pH_T_), partial pressure of carbon dioxide (pCO_2_), bicarbonate (HCO${}_{3}^{-2}$) and carbonate (CO${}_{3}^{-}$) concentrations, and aragonite saturation (Ω_arag_).

	**Piggy bank**	**T1**	**T2**	**T3**
Depth (m)	298	–:–	–:–	–:–
Temp (°C)	8.89 ± 0.6	9.59 ± 0.41	9.55 ± 0.28	10.11 ± 0.75
A_T_ (µmol kg^−1^)	2,278 ± 3	2,282 ± 30	2,305 ± 66	2,184 ± 65
pH_T_	7.62 ± 0.01	7.89 ± 0.04	7.62 ± 0.06	7.64 ± 0.06
pCO_2_ (µatm)	1,109 ± 106	580 ± 8	1,159 ± 34	1,031 ± 32
HCO_3_^−2^ (µmol kg^−1^)	2,145 ± 12	2,037 ± 27	2,168 ± 64	2,042 ± 61
CO_3_^−^ (µmol kg^−1^)	54 ± 5	97 ± 2	55 ± 2	56 ± 2
Ω_arag_	0.81 ± 0.07	1.47 ± 0.03	0.83 ± 0.03	0.85 ± 0.03

### Experimental set-up and seawater chemistry manipulation

Experiments were performed between June 20th and July 29th, 2016 in a temperature-controlled cold-room (∼9.5 °C ) at Temple University (Table 1). To test the physiological response of *L. pertusa*, we used six (6) independent 55 L tanks where the seawater chemistry was manipulated via CO_2_ additions using commercially available CO_2_/pH controller system (American Marine Inc., PINPOINT pH Monitor). The system is composed of a pH controller attached independently to each tank, which is connected to a solenoid valve that automatically delivers the desired concentration of CO_2_ according to a pre-set pH_T_ value. The pH_T_ meters underwent a two-point Tris-HCl and AMP-HCl calibration weekly (Dickson, Sabine & Christian, 2007). During the time of the experiment, a 25% water change was performed every other day to ensure good seawater conditions in the recirculating tanks. The water used in this experiment consisted of synthetic seawater (B-ionic^®^—ESV products) from which we were able to mimic the composition and total alkalinity of the *in situ* seawater chemistry. The experimental design consisted of two different pH_T_/Ω_arag_ target treatments (7.60/0.8 and 7.90/1.5), and two different total alkalinity values (2,200 and 2,300 µmol kg^−1^), which match *in situ* conditions for this species in the California margin (Table 1). Total alkalinity was only manipulated within the pH_T_/Ω_arag_ 7.60/0.8 treatment due to space and CO_2_-system restrictions. Total alkalinity was manipulated by adjusting the proportions of the different components of the custom-made artificial seawater. Logistically, we were unable to fully replicate our experiment at the tank level, thus our experimental design consisted of two tanks per treatment, with four fragments per tank. We used two (2) tanks with favorable pH/Ω_arag_ at 2,300 µmol kg^−1^ A_T_, two (2) tanks with unfavorable pH/Ω_arag_ at 2,300 µmol kg^−1^ A_T_, and two (2) tanks with unfavorable pH/Ω_arag_ at 2,200 µmol kg^−1^ A_T_ which approximates in situ conditions.

The pH_T_ of the experimental tanks was gradually brought down to the desired treatment conditions at a rate of ∼0.1 pH unit day^−1^. Total alkalinity (A_T_) was measured three times a week by acid-titration (0.1 mol L^−1^ HCl) on an open-cell potentiometric autotitrator (Mettler-Toledo DL15). The autotitrator underwent a three-point pH calibration weekly (NBS, National Bureau of Standards scale), and certified reference material (CRM) for A_T_ was measured weekly to ensure the accuracy of titrations (Batch 141; Dickson Labs, Seattle, WA, USA), which were always within ±1% error. Salinity was measured daily using a handheld refractometer (Vital Sine™), and temperature was recorded continually using a temperature logger (Onset HOBO Pendant^®^). CO2calc (Robbins et al., 2010) was used to calculate [pCO_2_], [HCO_3_^−^], [CO_3_^2−^], and Ω_arag_ using pH_T_, A_T_, salinity and temperature as input variables with the dissociation constants for boric acid and K_1_ and K_2_ from Leuker, Dickson & Keeling (2000), KHSO_4_from Dickson (1990), total boron from Lee et al. (2000) and pH on the total scale (pH_T_).

### Physiological measurements

#### Net Calcification

Net calcification was determined with the buoyant weighing technique originally described by Jokiel, Maragos & Franzisket (1978). A Denver Instruments SI-64 scale with a precision of 0.1 mg was used for this purpose. Briefly, the scale was mounted on an enclosed acrylic chamber fitted with a sliding panel that prevented air movement during weighing. By means of a tungsten wire, fragments of *L. pertusa* were weighed at the beginning and at the end of the three-week exposure, hanging under the scale and immersed in a seawater bath. Fragments were never exposed to air in any of the different measurements, and each one was weighed in triplicate to account for the variability in the measurement and the scale. Salinity and temperature of the water bath at the time of the buoyant weight were kept constant at 35 psu and 8.5 °C and were used to calculate the density of the medium in order to get the standardized dry-weight for net calcification. All fragments from each treatment were weighed when the pH reached the desired level and after 21 days. The standardized dry weight of each fragment (*W*_*a*_) was calculated using the following formula:

\begin{eqnarray*}Wa={W}_{w}/(1-({D}_{w}/SD)) \end{eqnarray*}

where *W*_*w*_ is the measured buoyant weight, *D*_*w*_ is the density of the seawater during measurement and *SD* is the coral skeletal density (2.62 g cm^−3^). Net calcification (*N*_*t*_) of *L. pertusa* was calculated as the change in weight over 3-week interval and is expressed as % d^−1^. Net calcification was calculated by the equation:

\begin{eqnarray*}{N}_{t}=100\times (({W}_{w2}-{W}_{w1})/({W}_{w1}({T}_{2}-{T}_{1}))) \end{eqnarray*}

where *W*_*w*2_ and *W*_*w*1_ are the final and initial standardized buoyant weights respectively, and *T*_1_ and *T*_2_ equal time 1 and time 2, respectively.

Skeletal density of coral fragments was obtained following a modified protocol proposed by Bucher, Harriott & Roberts (1998) in 22 coral fragments from the collection site. Briefly, fragments were initially soaked in 10% bleach for three days in order to remove living tissue. After this time, they were transferred to distilled water and left for 4 weeks to displace trapped air present in the skeletal voids. During this step, the container in which the fragments were held was tapped daily to assist bubbles removal (Bucher, Harriott & Roberts, 1998). At the end of period, no bubbles were observable when tapping the container. Each fragment was buoyant-weighed in distilled water, oven dried for 24 h at 60 °C and then dry-weighted using a Mettler Toledo scale model AB104-S with a precision of 0.1 mg. Skeletal density was obtained with the following formula:

\begin{eqnarray*}SD={D}_{w}/(1-({W}_{w}/{W}_{a})) \end{eqnarray*}

where *SD* = skeletal density, *D*_*w*_ = density of weighing medium, *W*_*w*_ = buoyant weight, and *W*_*a*_ = dry weight of coral skeleton (Jokiel, Maragos & Franzisket, 1978).

#### Feeding rates

Feeding rates were determined for each of the coral fragments as capture rate of freshly hatched *Artemia salina* nauplii (∼0.5 mm in length) over 1-hour interval. Experiments were conducted in a 0.8 l circular acrylic chamber with a stir bar in the bottom (all trials were set to slow flow of about 2–3 cm s^−1^). The chamber was placed inside a water bath that helped to maintain a constant temperature and set on top of a magnetic plate. Fragments from each tank were starved for 24 h before each experimental trial and feeding rates were assessed independently for each coral fragment (4 fragments per tank). An individual coral fragment was placed inside the acrylic chamber that was filled with seawater from the same treatment tanks and left for 30 min before starting the trial. The starting density of *A. salina* was 128 ± 3 *Artemia* l^−1^ (mean ± SD). At the end of the incubation period, the seawater from the chamber was filtered and the *A. salina* were counted under the dissecting scope. Feeding rates were standardized to number of polyps and are reported as number of prey polyp^−1^ h^−1^.

### Data analysis

The response of *Lophelia pertusa* grown under different pH/Ω_arag_ levels was obtained from the calcification and feeding rates. The effects of CO_2_ were analyzed in an ANOVA model with pH/Ω_arag_ levels as fixed factor (two levels). Replicate tanks were treated as random effects nested within pH/Ω_arag_ levels in order to test for possible tank effects. Since the tank factor was not significant for both response variables (*P* > 0.25), data from replicate tanks were pooled, thus individual fragments were analyzed as replicates (*n* = 8 per treatment) (Underwood, 1997). Shapiro–Wilk test was used to check for normality (*P* = 0.21) and Bartlett test for homogeneity of variances (*P* = 0.06). Results were considered statistically significant at a *P* < 0.05.

## Results

### Carbonate chemistry and coral distribution in the *in-situ* collection

**Figure 2:**
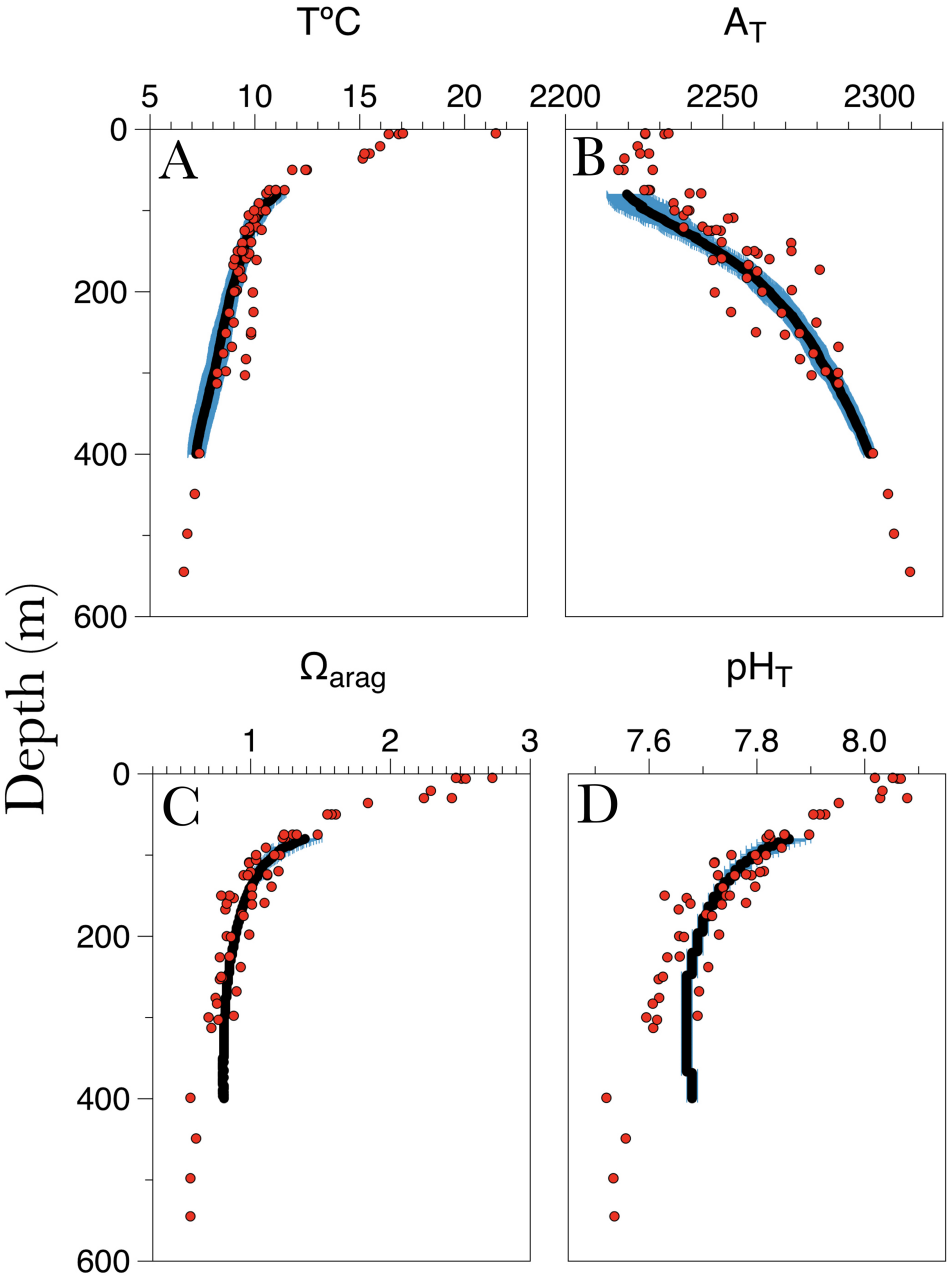
Water column profiles for (A) temperature (T °C), (B) total alkalinity (A_T_), (C) aragonite saturation (Ω_arag_) and (D) pH_T_(total scale) in the study site plotted against depth. Solid line indicates the average (±SD) of the seawater chemistry approximated from the CalCOFI database spanning a whole year from Nov 2014 to Nov 2015 the red dots represent the seawater chemistry from discrete water samples taken in the area of the collection sites. pH${}_{\mathrm{T}}^{\mathrm{es}}$ and A${}_{\mathrm{T}}^{\mathrm{est}}$ where obtained from the empirical model proposed by Alin et al. (2012) for the South Atlantic Bight for pH_T_ and A_T_.

**Figure 3:**
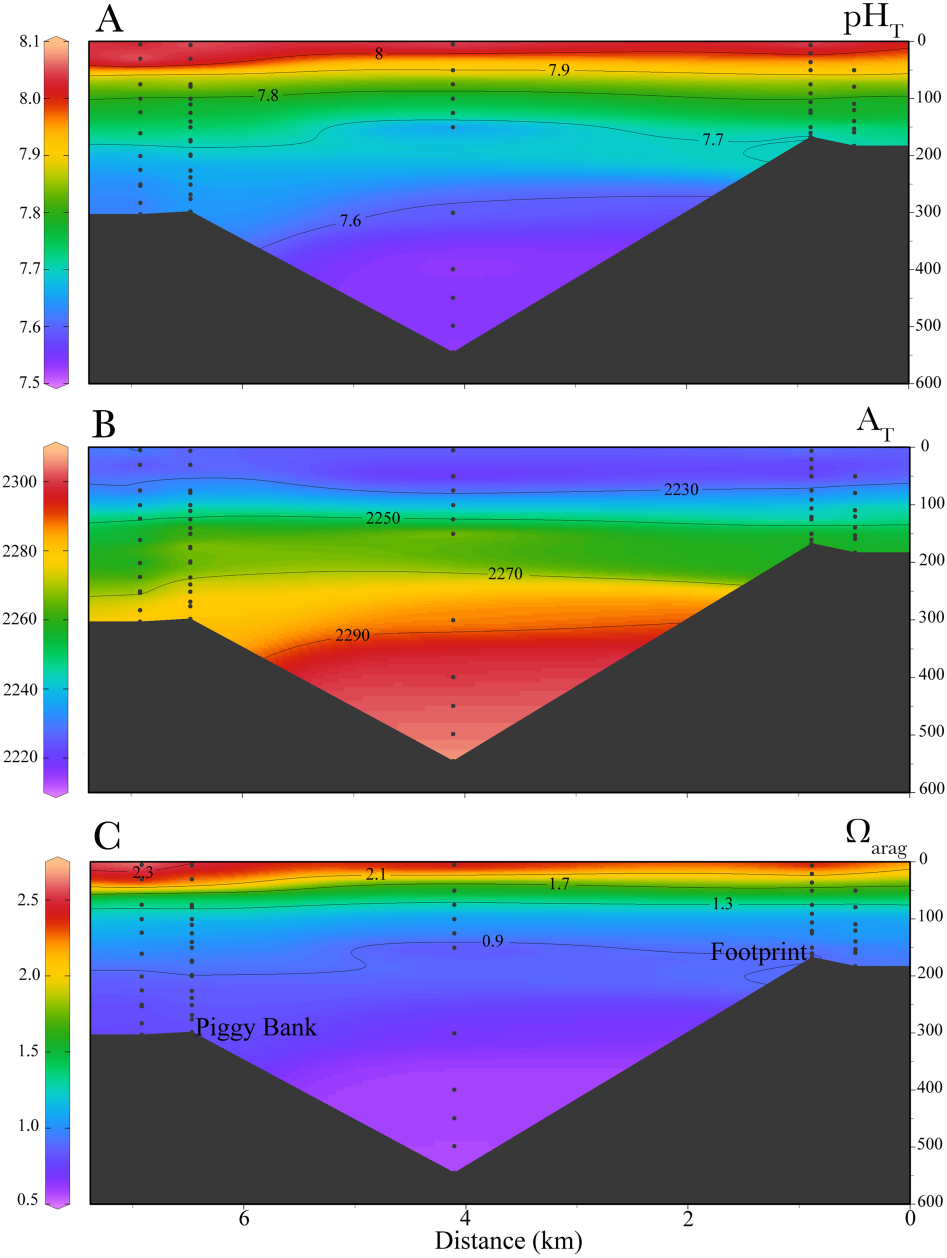
Cross-sectional profiles of *in-situ* carbonate chemistry parameters. Profiles are for (A) total pH (pH_T_), (B) total alkalinity (A_T_) (C) and aragonite saturation (Ω_arag_) in the South California Bight where *L. pertusa* were collected.

*L. pertusa* in the Piggy Bank and Footprint areas span a range of depths between 95 m and 296 m with a mean depth of 230 ± 73 m (Wickes, 2014). The *in situ* carbonate chemistry obtained from the targeted CTD-casts and the values approximated from the temperature, oxygen, and salinity from the CalCOFI database revealed low pH and aragonite saturation states year-round (Fig. 2). Aragonite saturation estimated from niskin bottle water sampling ranged from ∼0.6 (545 m) to ∼2.5 (surface 5 m), while for the data approximated from CalCOFI values ranged from ∼0.81 (400 m) to ∼1.4 (80 m). Average temperature, pH and Ω_arag_ at the site of sample collection (∼300 m) were 8.77 ± 0.62 °C , 7.62 ± 0.03 and 0.81 ± 0.07 respectively, while conditions close to the collection site as approximated from the CalCOFI data set were 8.06 ± 0.47 °C , 7.67 ± 0.01 and 0.81 ± 0.01 respectively (Table 2). Total alkalinity (A_T_) ranged from 2,285 (300 m) to 2,225 (80 m). The depth of the aragonite saturation horizon near the collection sites was at approximately 120 m depth (Fig. 3).

**Table 2:** Summary table of the CalCOFI physical-chemical conditions at 300 m depth. CalCOFI ancillary stations that were used to complement the characterization of the area around *L. pertusa* collection. Values for temperature (T °C), salinity (Sal), estimated pH on total scale (pH${}_{\mathrm{T}}^{\mathrm{es}}$), estimated total alkalinity (A${}_{\mathrm{T}}^{\mathrm{es}}$) and aragonite saturation (Ω_arag_) represent those found at 300 m depth (estimated values according to Alin et al., 2012). Line and group station refer to the CalCOFI original grid code, with the relative distance to *L. pertusa* collection. Values are given as mean ± SD.

						Physical—Chemical conditions at 300 m depth				
	Group station	Latitud	Longitude	Depth range (m)	Distance to collection site (km)	T°C	Sal	pH${}_{\mathrm{T}}^{\mathrm{es}}$	A${}_{\mathrm{T}}^{\mathrm{es}}$	Ω_arag_
Line 83.3	42	34°10′N	119°30′W	80–100	27	–:–	–:–	–:–	–:–	–:–
	51	33°52′N	120°8′W	80–100	58	–:–	–:–	–:–	–:–	–:–
	55	34°44′N	120°24′W	80–400	85	8.14 ± 0.02	34.21 ± 0.006	7.66 ± 0.0004	2286 ± 0.82	0.84 ± 0.001
	60	33°34′N	120°45′W	80–400	118	7.47 ± 0.03	34.11 ± 0.002	7.68 ± 0.0002	2279 ± 0.50	0.85 ± 0.002
Line 86.7	35	33°49′N	118°37′W	80–400	76	8.77 ± 0.01	34.23 ± 0.01	7.65 ± 0.003	2282 ± 0.84	0.84 ± 0.004
	40	33°39′N	118°58′W	80–400	56	8.41 ± 0.02	34.23 ± 0.005	7.65 ± 0.0004	2285 ± 0.61	0.83 ± 0.001
	45	33°29′N	111°19′W	80–400	51	8.14 ± 0.03	34.21 ± 0.002	7.66 ± 0.0005	2285 ± 0.01	0.84 ± 0.004
	55	33°09′N	120°01′W	80–400	94	7.79 ± 0.04	34.19 ± 0.002	7.67 ± 0.0006	2286 ± 0.25	0.84 ± 0.001
	60	32°59′N	120°20′W	80–400	131	7.40 ± 0.02	34.07 ± 0.006	7.69 ± 0.0009	2277 ± 0.99	0.86 ± 0.001

### Experimental conditions

All of the measured variables, including the seawater chemistry, were kept relatively constant through the experiment with small variations due to tank maintenance and water changes (Table 1). Physical and chemical conditions were similar to the *in situ* seawater chemistry where *L. pertusa* is distributed in the South California Bight.

### Calcification rates

**Figure 4:**
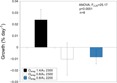
Growth (% day^−1^) of *L. pertusa* fragments in the different treatment conditions. Aragonite saturation (Ω_arag_) and total alkalinity (A_T_) treatments used in the study. Values are reported as mean ± SD (*n* = 8).

We found 100% survival of *L. pertusa* corals (*n* = 24 fragments) in the different treatments studied. The corals still appeared to be in relatively good condition, as indicated by their extended polyps. On average, corals grown in the favorable treatment (pH_T_ = 7.90, Ω_arag_ = 1.5) calcified at a rate of 0.02 ± 0.009% day^−1^ (mean ± SD), while for the corals grown in the unfavorable treatment (pH_T_ = 7.60, Ω_arag_ = 0.8), the value was −0.010 ± 0.014% day^−1^ (mean ± SD). Corals grown in the unfavorable treatment with low A_T_ calcified at a similar rate to the corals grown in the unfavorable treatment with high A_T_, with net calcification rates of −0.008 ± 0.006% day^−1^ (mean ± SD). We found a significant negative effect of pH on net calcification (ANOVA *F*_2,20_ = 25.17 *P* < 0.001) where corals grown in the unfavorable treatments experienced a decreased calcification rate with net dissolution of the skeleton by the end of the three-week period (Fig. 4, Table 3). However, there was no significant difference between A_T_ treatments at low pH.

Skeletal density from the 22 fragments measured were in average 2.62 ± 0.28 g cm^−3^ (mean ± SD) with a range between 1.83 and 2.83 g cm^−3^. There was a good linear relationship between the buoyant weight and dry weight measurements taken for the calculations of the skeletal density (*R*^2^ = 0.98).

**Table 3:** Summary of the response variables. Summary of the results from the response variables used to measure ocean acidification effect on fragments of *L. pertusa* in the different experimental conditions of aragonite saturation (Ω_arag_) and total alkalinity (A_T_). Values for net calcification (G % d^−1^) and feeding behavior (capture rates) are given as mean ± SD.

Treatment	*N*	Initial skeletal weight	Polyp #	G (% d^−1^)	Capture rates
Ω_arag_ 1.4/A_T_2,300	8	9.69 ± 2.42	11 ± 4	0.0238 ± 0.009	4.09 ± 1.41
Ω_arag_ 0.8/A_T_ 2,300	8	11.56 ± 3.60	10 ± 5	−0.0102 ± 0.014	2.65 ± 1.99
Ω_arag_ 0.8/A_T_ 2,200	7	10.95 ± 4.24	9 ± 5	−0.0085 ± 0.006	3.86 ± 4.36

### Feeding rates

**Figure 5:**
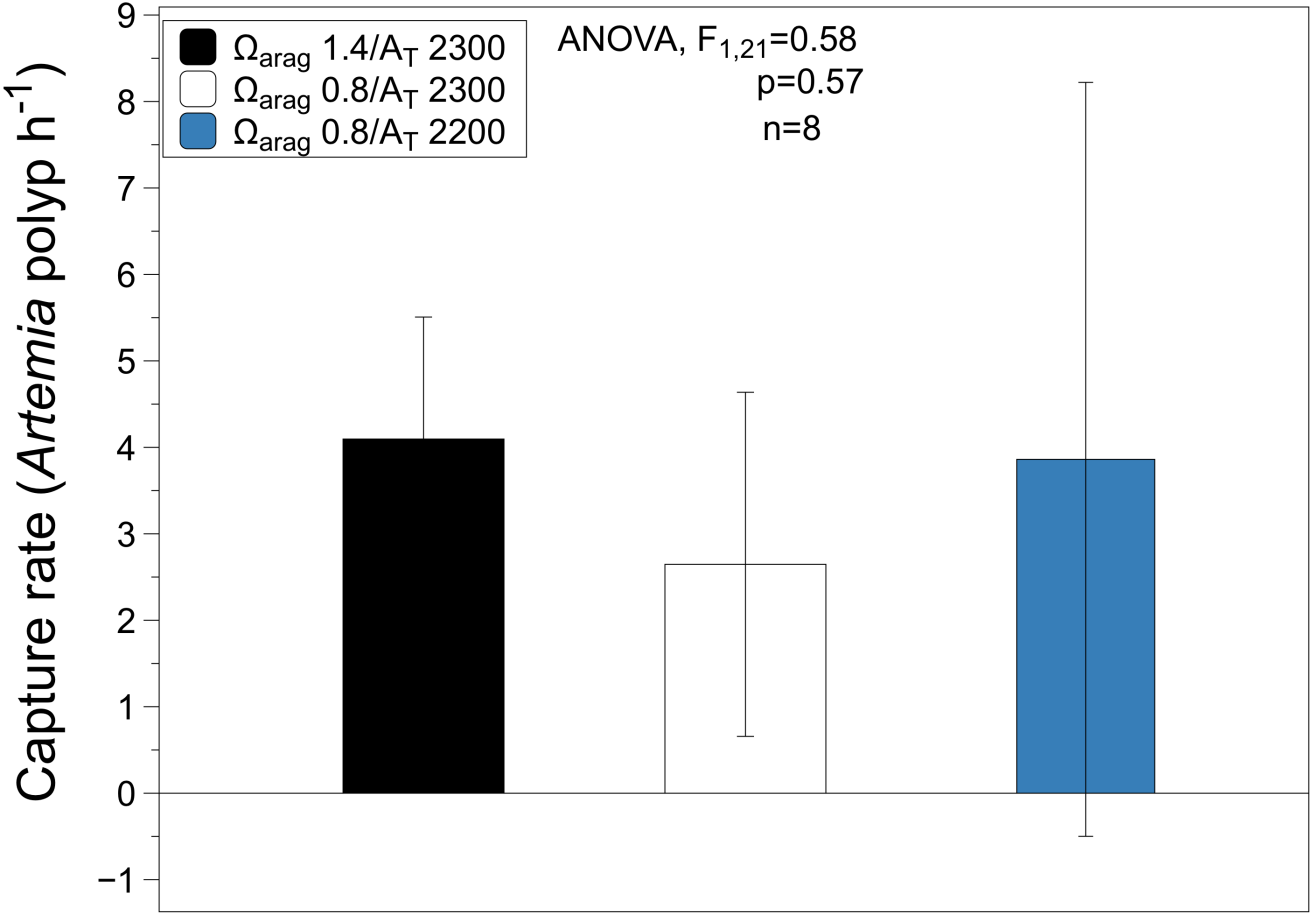
Average capture rates of *Artemia* standardize per polyp per hour. Aragonite saturation (Ω_arag_) and total alkalinity (A_T_) treatments used in the study. Values are reported as mean ± SD (*n* = 8).

The average capture rate of *Artemia* in the favorable treatment were 4 ± 1 *Artemia* polyp^−1^ h^−1^ (mean ± SD) with a range of values between 2–6 *Artemia* polyp^−1^ h^−1^. For the unfavorable treatment, the average capture rate was 3 ± 2 *Artemia* polyp^−1^ h^−1^ (mean ± SD) with a range values between 1–7 *Artemia* polyp^−1^ h^−1^. The same pattern was observed for the capture rates in the low alkalinity treatment, which were similar to the other treatments with an average of 4 ± 4 *Artemia* polyp^−1^ h^−1^ (mean ± SD) and a range value between 1 and 14 *Artemia* polyp^−1^ h^−1^ (Fig. 5, Table 3). There were no significant differences for the capture rate of *Artemia* between treatments (ANOVA *F*_2,21_ = 0.58, *P* = 0.57).

## Discussion

This study shows that populations of *L. pertusa* from the South California Bight (SCB) are already experiencing negative effects of low pH and Ω_arag_. These suboptimal conditions result in a decrease in net calcification rates in the laboratory. These experiments were carried out under the conditions it normally experiences year-round, compared to the typical conditions in other parts of its range. Calcification rates found in the present study for favorable conditions are in accordance with those found in other studies performed with *L. pertusa* under similar conditions (pH 7.9, Ω_arag_ 1.3, A_T_ ∼2,300) (Maier et al., 2009; Hennige et al., 2014; Lunden et al., 2014; Georgian et al., 2016a). However, the negative net calcification of −0.0102 and −0.008 G (% d^−1^) for the low pH/ Ω_arag_ treatments are unexpected. Given the conditions in which this species grows in the SCB (pH ∼7.6 and Ω_arag_ ∼0.8), we expected positive calcification rates of coral fragments grown in the unfavorable treatment. Negative net calcification rates have been also found for *L. pertusa* from the Gulf of Mexico under similar experimental conditions (Lunden et al., 2014; Georgian et al., 2016a; Kurman et al., 2017), however these populations live in Ω_arag_ >1 and do not experience undersaturation yet (Georgian et al., 2016b). It has been documented that *L. pertusa* populations from the North Atlantic seems to be more resistant to OA effects, at least in the near future where the Ω_arag_ levels fall near the saturation horizon or slightly undersaturated (Form & Riebesell, 2012; Büscher, Form & Riebesell, 2017). We found that 100% of the coral fragments exhibited a net loss of skeleton in the acidified treatment, rendering an important concern of the fate of these scleractinians and the communities they support on the California Margin.

The findings of this study are in broad agreement with other investigations of the effects of ocean acidification on *L. pertusa*, although a wide variety of responses have been shown. Kurman et al. (2017) found a negative response for coral fragments from the Gulf of Mexico at the end of a 6-month period under saturation states of 0.8, and by the end of the experiment, 100% of the coral fragments showed negative calcification. However, some genotypes in this study were capable of maintaining positive net calcification significantly longer than others. In a short-term experiment using corals from these same populations, Lunden et al. (2014) found different responses among genotypes, with a change in the overall response around a pH of 7.75, corresponding to a saturation state of almost exactly 1. The majority of studies that have found no effect in decreasing aragonite/pH have used levels only slightly undersaturated (∼1) or still above saturation (>1) (Maier et al., 2012; Movilla et al., 2014; Büscher, Form & Riebesell, 2017). Maier et al. (2016) found negative calcification rates for *Madrepora oculata* only when the Ω_arag_ fell below a threshold of 0.9. Therefore, it is likely that the negative effects on cold-water corals are only evident when Ω_arag_ falls well below a certain threshold, and the erosive forces outweigh the accretionary forces and the calcification rate of *L. pertusa* colonies cannot keep up with the increasing rate of skeletal dissolution.

The absence of reef-framework and low proportion of live coral in the SCB has been attributed to the low saturation states of the region (Wickes, 2014). However, it is known that CWCs exert a strong biological control on calcification rates, elevating and or modifying the carbonate chemistry in the compartments were the calcification occurs, suggesting that CWCs are still able to produce calcium carbonate at low pH/ Ω_arag_ (McCulloch et al., 2012; Raybaud et al., 2017). Raybaud et al. (2017) provided a numerical framework of internal carbonate chemistry in several species of scleractinian corals, and they found that CWCs exert the strongest control on Ω_arag_, elevating carbonate concentrations up to 10 times that of the surrounding seawater. The finding here of an independence of calcification rate on total alkalinity in the unfavorable treatment suggests that the control of Ω_arag_ is by factors other than negative ion transport, such as proton pumps controlling internal pH.

Due to the demonstrated ability of CWCs to calcify at reduced Ω_arag_, it may be that dissolution rates are the most important variable to take into consideration when determining overall net calcification for *L. pertusa* living at undersaturation. This rate will primarily be a function of the Ω_arag_ but also the relative degree of tissue coverage of the skeleton (i.e., Gammon et al., 2018). Where there is more exposed skeleton, there will be higher dissolution. This could partially explain the lower skeletal density that was measured in the corals from this study (2.62 g cm^−3^) as compared to the corals in the Gulf of Mexico (2.81 g cm^−3^, Lunden, Georgian & Cordes, 2013). Gross calcification and tissue coverage were not monitored in the present study, and therefore it is difficult to conclude whether the pattern observed is due to higher dissolution rates alone or if there is also a decline in the gross calcification rate.

In this study, feeding performance (capture rate) was similar between treatments, suggesting that pH/Ω_arag_ did not compromise the ability of a given coral nubbin to obtain food. These results contrast with other studies where *L. pertusa* can either increase feeding and respiration rates to meet the elevated energetic challenges of low pH and Ω_arag_, or decrease feeding and respiration to undergo metabolic depression to presumably wait for a return to favorable conditions (Houlbrèque et al., 2015; Georgian et al., 2016a). Our results of prey capture (3–4 *Artemia* polyp^−1^ h^−1^) fall within the lower range for some studies, i.e., *Lophelia pertusa* from the North Atlantic (Purser et al., 2010: 6 ± 1 *Artemia* polyp^−1^ h^−1^; Orejas et al., 2016: 22 ± 8 *Artemia* polyp^−1^ h^−1^; Georgian et al., 2016a: 8 ± 1 *Artemia* polyp^−1^ h^−1^) and within the higher range for populations from the Gulf of Mexico (Georgian et al., 2016a: 2 ± 1 *Artemia* polyp^−1^ h^−1^). The higher values normally found for the North Atlantic populations are related to the apparent higher metabolic rates in those populations (Purser et al., 2010; Orejas et al., 2016).

Food availability and nutrient supply are known to be important in shallow-water tropical systems, where additional inputs generate the extra energy required for calcification under ocean acidification (Cohen & Holcomb, 2009; Houlbrèque et al., 2015). On the other hand, some studies performed in CWCs have found that, in general, corals grown under higher food concentrations do not increase calcification rates as compared to low food concentrations (Maier et al., 2016; Büscher, Form & Riebesell, 2017), even though deep-water corals are heterotrophic organisms that rely entirely on external food supplies. Still, it is plausible that the high productivity of the California Current System, along with *L. pertusa’* s relatively shallow distribution in the area can explain why this coral population persists under the low pH and saturation state that it experiences there, although at the expense of skeletal density and framework stability. However, it is not known if these coral populations are actively growing at this time or are relics of populations that existed before the onset of ocean acidification, since the industrial revolution in the 19 century. It is important to point out that while experimental conditions in controlled systems are set-up to mimic the natural environment as close as possible, it is specially challenging to recreate the full spectrum of conditions, especially the variable food supply and nutrient availability typical of the bathyal environment.

*L. pertusa* is normally associated with high-energy environments, due to enhanced food supply from the surface via advection, and resuspension from internal waves and currents (Davies et al., 2009; Roberts et al., 2009). It has been shown that high surface primary productivity together with the bentho-pelagic coupling affects the structure of the benthic fauna, including increasing deep-sea coral diversity (Davies et al., 2008; Jansen et al., 2018). Jansen et al. (2018) found a positive correlation between the abundance of deep-water suspension feeders (coral and sponges) and high surface primary productivity, which ultimately sinks and provides an important source of particulate organic carbon (POC). Similar patterns have been found in the North Atlantic (Lacharité & Metaxas, 2017) and in the Tasmanian seamounts where the abundance of the scleractinians *Solenosmilia variabilis* and *Enallopsammia rostrata* between 750–1,400 m, with peak distribution at or slightly below the ASH, can be explained by the high input of marine snow and particulate organic matter in this area (Thresher et al., 2011). Similarly, these species have been found in comparable conditions in the North Central Pacific, also associated with areas of high input of nutrients and productivity at the surface (Baco et al., 2017). Coral calcification is energetically costly and can consume up to 20% of the coral’s energy budget (Cohen & Holcomb, 2009). This requirement can increase by 20–30% under ocean acidification scenarios expected by the end of the century (McCulloch et al., 2012).

In addition to ocean acidification, climate and ocean change are expected to alter primary productivity in coastal regions, as well as the rate of export to deep waters. Increasing stratification due to the more rapid rise of temperature in surface waters will reduce the export of POC to depth and the delivery of deep-water nutrients to the surface (Palacios et al., 2004). However, there is some evidence that increased shore wind velocity can lead to increased nutrient delivery in upwelling systems (Bakun et al., 2015; Wang et al., 2015), which if coupled with an efficient feeding behavior, CWCs from California margin might be able to increase their energy intake for metabolic function in order to keep the homeostatic control. Nevertheless, this increase in nutrients in the CCS might be counteracted by the decrease in oxygen concentration (−18%) and pH (−0.5 units) projected for this area by the end of the century (Rykaczewski & Dunne, 2010). *Lophelia pertusa* colonies cannot survive under low oxygen conditions based on lab experiments, although the exact limit of their tolerance appears to vary by population (<3.4 ml l^−1^, Dodds et al., 2007; <1.5 ml l^−1^, Lunden et al., 2014). However, further multi-stressor experiments are necessary to determine how all of these factors interact and affect *L. pertusa*. But it is clear that the outcome for specific populations is a combination of their genetic variability along with their history of food availability and therefore energetic reserves, which will result in different physiological responses to the exacerbated challenges that deep-water corals will face in the future.

## Conclusions

These results highlight that *L. pertusa* is persisting along the California margin under more extreme carbonate chemistry conditions than have been found in other populations. Although our results show decreased net calcification under unfavorable Ω_arag_, it is important to bear in mind that experimental conditions are normally tightly controlled, which does not account for the dynamics normally seen in natural conditions, especially in places with high productivity such as the California Current System. It is possible that the CCS provides an exception where an active growing population can be maintained at undersaturated conditions of calcium carbonate as a result of elevated food availability from surface layers. *L. pertusa* is an important habitat structuring species, forming habitat for many species, including some of commercial importance, in one of the most productive regions in the world (Barton et al., 2015). Consequently, the ecological impacts of ocean acidification on the deep-water corals of the California Current system are of significant concern given that this region will experience year-round undersaturation most of the water column within the next 20–30 years and by 2050.

##  Supplemental Information

10.7717/peerj.5671/supp-1 Table S1 Buoyant-weight data points for the different treatments Time 1 corresponds to the initial time when the first B-W measurement was made. Time 2 correspons to the final time measurement. It is provided in the table the raw buoyant-weight value as well as the standardize Dry weight value, which is the one taken into consideration for the analysis.

10.7717/peerj.5671/supp-2 Table S2 Capture rate data points for the different treatments Each single entry corresponds to an individual fragment from the different tanks and treatment exposures. In this table, raw values for the initial Artemia density and final Artemia used to calculate the delta. Standardize values are provided as numbers of Artemia per polyp per hour **

10.7717/peerj.5671/supp-3 Figure S1 Values for pH_T_ during the experimental trial Each dot corresponds to the average pH_T_ taken per day. Open circles correspond to the obtained pH_T_ in the acidified conditions and closed circles correspond to the pHT in the non-acidified conditions

10.7717/peerj.5671/supp-4 Supplemental Information 1 Aragonite saturation during the experimental trial Each data point indicates a discrete value of the aragonite saturation at each interval point. Black dots indicate samples collected in the non-acidified “favorable condition” treatment, while white dots indicate samples collected in the acidified “unfavorable condition” treatment. Aragonite saturation was obtained from the pH_T_ and the A_T_ and computed in CO2calc.

## Additional Information and Declarations

### Field Study Permissions

The following information was supplied relating to field study approvals (i.e., approving body and any reference numbers): Research was conducted under permit CINMS-2015-002 issued Feb 4, 2015 by NOAA Office of National Marine Sanctuaries, in support of the project “Climate Vulnerability Assessment for Deep-Sea Coral Ecosystems in California”.

### Data Availability

The following information was supplied regarding data availability: The raw data are provided in the Supplemental Files.
