# Supplementary figures and images for: Growth and feeding of deep-sea coral Lophelia pertusa from the California margin under simulated ocean acidification conditions
Source: PeerJ. 2018 Sep 27;6:e5671. doi: 10.7717/peerj.5671 (PMC6164558; doi:10.7717/peerj.5671)

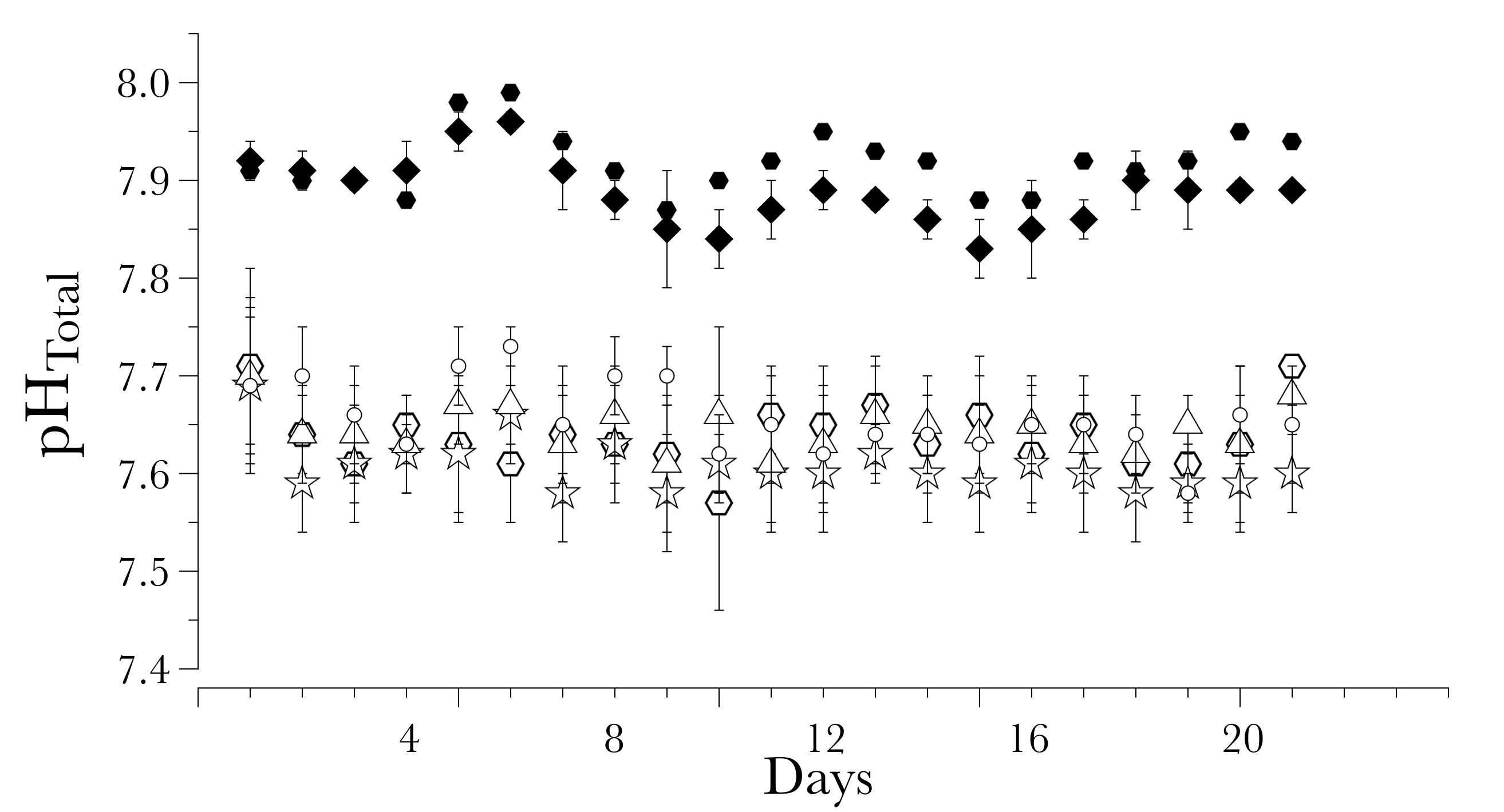

Supplement: Figure S1 — Each dot corresponds to the average pHT taken per day. Open circles correspond to the obtained pHT in the acidified conditions and closed circles correspond to the pHT in the non-acidified conditions [file peerj-06-5671-s003.png]

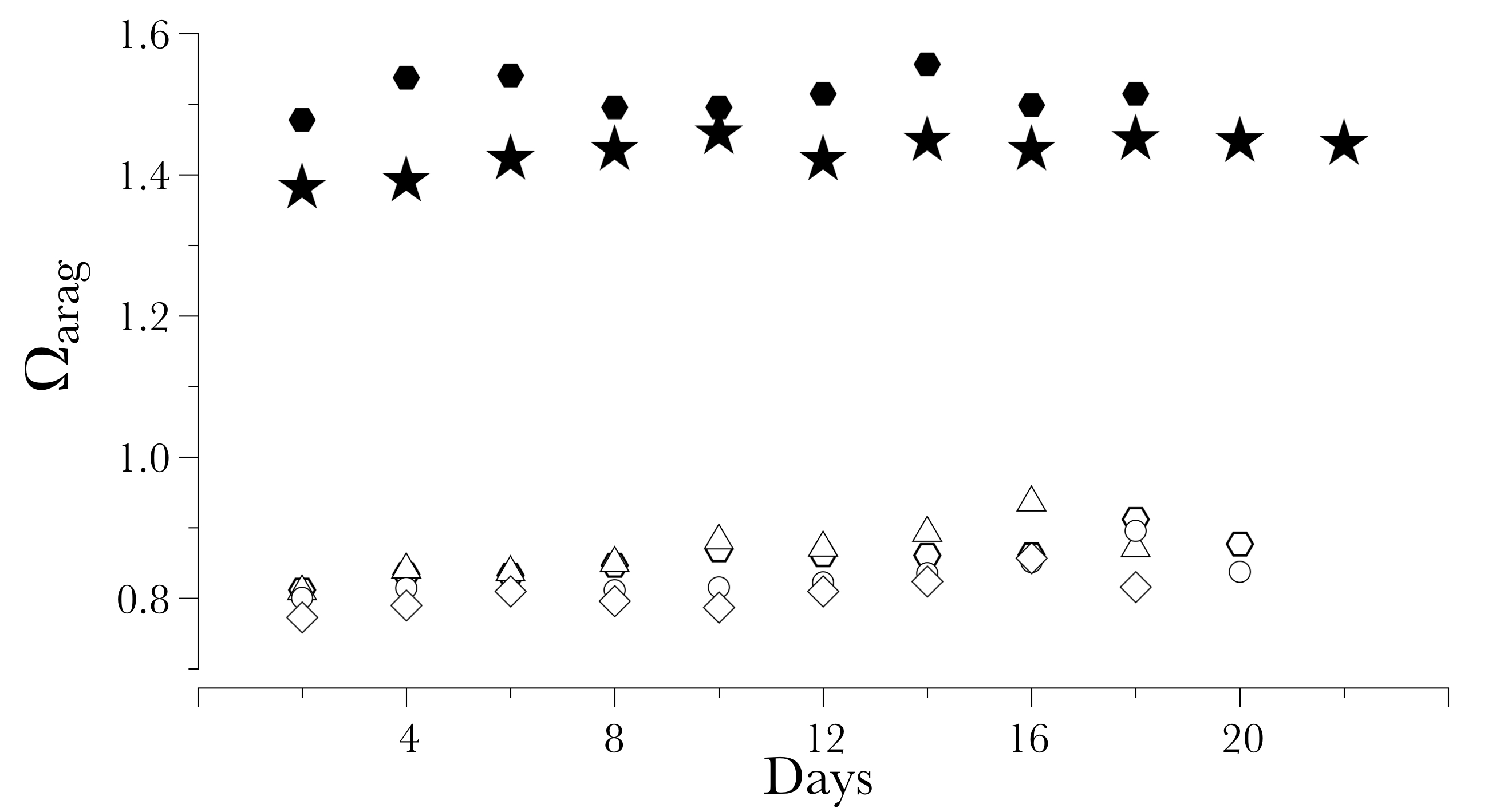

Supplement: Supplemental Information 1 — Each data point indicates a discrete value of the aragonite saturation at each interval point. Black dots indicate samples collected in the non-acidified “favorable condition” treatment, while white dots indicate samples collected in the acidified “unfavorable condition” treatment. Aragonite saturation was obtained from the pHT and the AT and computed in CO2calc. [file peerj-06-5671-s004.png]
